# Renal dysfunction, malignant neoplasms, atherosclerotic cardiovascular diseases, and sarcopenia as key outcomes observed in a three-year follow-up study using the Werner Syndrome Registry

**DOI:** 10.18632/aging.204681

**Published:** 2023-05-01

**Authors:** Yukari Maeda, Masaya Koshizaka, Mayumi Shoji, Hiyori Kaneko, Hisaya Kato, Yoshiro Maezawa, Junji Kawashima, Kayo Yoshinaga, Mai Ishikawa, Akiko Sekiguchi, Sei-Ichiro Motegi, Hironori Nakagami, Yoshihiko Yamada, Shinji Tsukamoto, Akira Taniguchi, Ken Sugimoto, Yoichi Takami, Yukiko Shoda, Kunihiko Hashimoto, Toru Yoshimura, Asako Kogure, Daisuke Suzuki, Naoki Okubo, Takashi Yoshida, Kazuhisa Watanabe, Masafumi Kuzuya, Minoru Takemoto, Junko Oshima, Koutaro Yokote

**Affiliations:** 1 Department of Endocrinology, Hematology, and Gerontology, Chiba University Graduate School of Medicine, Chiba, Japan; 2 Department of Metabolic Medicine, Faculty of Life Sciences, Kumamoto University, Kumamoto, Japan; 3 Department of Dermatology, Gunma University Graduate School of Medicine, Maebashi, Japan; 4 Department of Health Development and Medicine, Osaka University Graduate School of Medicine, Osaka, Japan; 5 Department of Medicine, Division of Diabetes, Metabolism and Endocrinology, Atami Hospital, International University of Health and Welfare, Atami, Japan; 6 Department of Orthopaedic Surgery, Nara Medical University, Nara, Japan; 7 General Geriatric Medicine, Kawasaki Medical School, Okayama, Japan; 8 Department of Geriatric and General Medicine, Osaka University Graduate School of Medicine, Osaka, Japan; 9 Department of Dermatology, Sumitomo Hospital, Osaka, Japan; 10 Department of Endocrinology and Metabolic Medicine, Nippon Life Hospital, Osaka, Japan; 11 Diabetes and Endocrinology, Saga-Ken Medical Centre Koseikan, Saga, Japan; 12 Department of Dermatology, Showa General Hospital, Tokyo, Japan; 13 Department of Orthopaedics, Graduate School of Medical Science, Kyoto Prefectural University of Medicine, Kyoto, Japan; 14 Department of Orthopaedic Surgery, North Medical Center, Kyoto Prefectural University of Medicine, Kyoto, Japan; 15 Department of Community Healthcare and Geriatrics, Nagoya University Graduate School of Medicine, Nagoya, Japan; 16 Geriatric Medicine, Meitetsu Hospital, Nagoya, Japan; 17 Department of Medicine, Division of Diabetes, Metabolism and Endocrinology, International University of Health and Welfare, Narita, Japan; 18 Department of Laboratory Medicine and Pathology, University of Washington, Seattle, WA 98195, USA

**Keywords:** disease profile, long-term follow-up, malignant neoplasm, renal function, Werner syndrome

## Abstract

Werner syndrome is an adult-onset progeria syndrome that results in various complications. This study aimed to clarify the profile and secular variation of the disease. Fifty-one patients were enrolled and registered in the Werner Syndrome Registry. Their data were collected annually following registration. A cross-sectional analysis at registration and a longitudinal analysis between the baseline and each subsequent year was performed. Pearson's chi-squared and Wilcoxon signed-rank tests were used. Malignant neoplasms were observed from the fifth decade of life (mean onset: 49.7 years) and were observed in approximately 30% of patients during the 3-year survey period. Regarding renal function, the mean estimated glomerular filtration rate calculated from serum creatinine (eGFRcre) and eGFRcys, which were calculated from cystatin C in the first year, were 98.3 and 83.2 mL/min/1.73 m^2^, respectively, and differed depending on the index used. In longitudinal analysis, the average eGFRcre for the first and fourth years was 74.8 and 63.4 mL/min/1.73 m^2^, showing a rapid decline. Secular changes in Werner syndrome in multiple patients were identified. The prevalence of malignant neoplasms is high, and renal function may decline rapidly. It is, therefore, necessary to carry out active and detailed examinations and pay attention to the type and dose of the drugs used.

## INTRODUCTION

Werner syndrome is an autosomal recessive adult-onset progeroid disorder that affects approximately 700-2,000 individuals in Japan [1–4]. Patients with Werner syndrome present with various aging phenotypes from a young age. Graying and/or loss of hair presents in their third decade of life; bilateral cataracts and diabetes, around their fourth decade of life; and atherosclerotic diseases and malignant neoplasms, around their fifth decade of life [5]. These patients also develop a high proportion of skin ulcers, often requiring amputation of the lower extremities. Although it was previously reported that patients with Werner syndrome die at 46 years of age on average [6], recent evidence indicates the average age of death is 59 years [7]. In Werner syndrome, quality of life (QOL) and activities of daily living (ADL) decline due to the various symptoms [8].

Since it is a rare disease, there is often a long period between the disease onset and the diagnosis [9]. Therefore, early detection and therapeutic intervention are important. Recently, the number of long-term survivors has increased due to therapeutic advances; however, new complications have also been observed. Although detailed and long-term involvement in medical care is essential for maintaining QOL and ADL, few reports have followed the changes over time in patients with Werner syndrome.

The Werner Syndrome Registry was established in 2017 to investigate the disease, recruit participants for clinical trials, and provide information to patients and physicians. In this report, the updated cross-sectional and longitudinal analyses of the Werner Syndrome Registry database were performed to reveal the current status and natural course in patients with Werner syndrome.

## RESULTS

### Werner Syndrome Registry cross-sectional analysis

Twelve facilities and fifty-one diagnosed patients were enrolled in the registry. Table 1 shows the patients’ characteristics; the percentage of baseline major signs, clinical symptoms, and comorbidities in patients with Werner syndrome; the drugs administered for diabetes, dyslipidemia, and hypertension; and the blood examination results at the point of enrollment to the registry. The patients’ mean registered age was 49.0 ± 7.2 years. Although the mean age of onset, inferred from the interviews or medical histories, was 25.8 ± 9.2 years, the mean age at diagnosis was 41.8 ± 8.2 years. Patients with Werner syndrome were lower in height, weight, and body mass index (BMI) than the average Japanese adults [10, 11]. Despite having a low BMI, the mean waist circumference was 77.2 ± 11.1 cm, and the mean visceral fat area measured by computed tomography was 97.5 ± 57.5 cm^2^. The mean skeletal muscle index (SMI) on dual-energy X-ray absorptiometry was 5.36 ± 1.61 kg/m^2^ for men and 3.99 ± 0.98 kg/m^2^ for women, the grip strength (in the right hand) was 22.8 ± 8.6 kg for men and 12.9 ± 5.5 kg for women, and the average gait speed was 0.96 ± 0.56 m/sec. The grip strength and SMI met the diagnostic criteria for sarcopenia. Approximately 30% of patients’ parents had consanguineous marriages.

**Table 1:** Patients’ characteristics at baseline.

	***N* ^a^**	**% (*n* patients with this characteristic)**				
**Major signs**						
Graying and/or loss of hair	51	98.0 (50)	
Bilateral cataracts	51	100 (51)	
Skin changes	51	98.0 (50)	
Intractable skin ulcers	51	66.7 (34)	
Soft tissue calcification	49	93.9 (46)	
Bird-like face	51	92.2 (47)	
High-pitched voice	51	82.4 (42)	
**Clinical symptoms**						
Diabetes, IGT	51	68.6 (35)	
Dyslipidemia	51	66.7 (34)	
Hypertension	48	39.6 (19)	
Fatty liver	49	59.2 (29)	
Cerebral bleeding	51	0 (0)	
Cerebral infarction	51	0 (0)	
AP or MI	51	2.0 (1)	
PAD	51	11.8 (6)	
Malignant neoplasm	51	25.5 (13)	
Amputation	51	11.8 (6)	
**Medications**						
(1) For diabetes						
DPP-4 inhibitor	35	34.3 (12)	
Biguanide	35	34.3 (12)	
Thiazolidine	35	42.9 (15)	
Alpha GI	35	5.7 (2)	
Sulfonylurea	35	8.6 (3)	
Glinide	35	0 (0)	
SGLT2 inhibitor	35	2.9 (1)	
GLP-1 analog	35	5.7 (2)	
Insulin	35	14.3 (5)	
(2) For dyslipidemia						
Statin	34	58.8 (20)	
Fibrate	34	2.9 (1)	
Ezetimibe	34	0 (0)	
EPA	34	14.7 (5)	
Ion exchange resin	34	0 (0)	
Nicotinic acids	34	14.7 (5)	
(3) For hypertension, among others						
Ca blocker	19	47.4 (9)	
ARB	19	36.8 (7)	
ACE inhibitor	19	0 (0)	
Alpha1 blocker	19	0 (0)	
Beta blocker	19	10.5 (2)	
Diuretics	19	0 (0)	
Eplerenone	19	0 (0)	
Nitrate	48	0 (0)	
Aspirin	48	4.2 (2)	
Antiplatelet	47	10.6 (5)	
Warfarin	48	0 (0)	
Anticoagulant	47	2.1 (1)	
	**Total**		**Male**		**Female**	
	***N***	**Mean ± SD**	***N***	**Mean ± SD**	***N***	**Mean ± SD**
**Patients’ backgrounds**						
Registered age (years)	51	49.0 ± 7.2	27	48.3 ± 7.1	24	49.7 ± 7.5
Onset age (years)	41	25.8 ± 9.2	21	28.0 ± 8.1	20	23.4 ± 9.9
Diagnosed age (years)	50	41.8 ± 8.2	26	41.8 ± 6.0	24	41.8 ± 10.3
Consanguineous marriage (%)	14/48	29.2	7/24	29.2	7/24	29.2
**Physical findings/function**						
Height (cm)	51	154.2 ± 10.5	27	160.1 ± 8.2	24	147.6 ± 8.7
Average 40s Japanese height (cm)				171.5 ± 5.8		158.1 ± 5.4
Body weight (kg)	51	43.7 ± 9.6	27	49.6 ± 8.6	24	37.1 ± 5.5
Average 40s Japanese body weight (kg)				72.8 ± 12.8		55.6 ± 10.0
BMI (kg/m^2^)	51	18.3 ± 3.1	27	19.4 ± 3.2	24	17.1 ± 2.5
Average 40s Japanese BMI (kg/m^2^)				24.7 ± 4.0		22.3 ± 4.0
Systolic blood pressure (mmHg)	45	123 ± 19	23	128 ± 19	22	118 ± 18
Diastolic blood pressure (mmHg)	45	69 ± 13	23	73 ± 11	22	65 ± 13
Pulse (/min)	43	87 ± 14	22	88 ± 14	21	85 ± 13
Waist circumference (cm)	30	77.2 ± 11.1	17	80.2 ± 11.3	13	73.2 ± 9.9
Visceral fat area (cm^2^)	14	97.5 ± 57.5	6	117.3 ± 63.6	8	82.7 ± 51.7
Skeletal muscle mass index (kg/m^2^)	18	4.60 ± 1.44	8	5.36 ± 1.61	10	3.99 ± 0.98
Grip strength (right) (kg)	32	18.1 ± 8.8	17	22.8 ± 8.6	15	12.9 ± 5.5
Grip strength (left) (kg)	32	16.6 ± 7.8	17	21.0 ± 7.4	15	11.5 ± 4.6
Walking speed (m/sec)	16	0.96 ± 0.56	8	1.03 ± 0.60	8	0.88 ± 0.55
**Blood examinations**						
WBC (/μL)	50	7504 ± 2268	27	7486 ± 2605	23	7525 ± 1855
RBC (x10^4^/μL)	50	414 ± 85	27	429 ± 70	23	397 ± 99
Hb (g/dL)	50	12.6 ± 2.1	27	13.2 ± 2.1	23	12.0 ± 1.9
Plt (x10^4^/μL)	50	28.6 ± 9.1	27	26.0 ± 7.2	23	31.7 ± 10.2
AST (U/L)	51	31.6 ± 17.5	27	34.9 ± 19.6	24	27.8 ± 14.4
ALT (U/L)	51	41.2 ± 32.9	27	48.6 ± 38.2	24	33.0 ± 23.9
γGTP (U/L)	49	98.3 ± 103.6	27	95.1 ± 96.4	22	102.2 ± 114.1
LDH (U/L)	48	223 ± 161	27	234 ± 208	21	210 ± 65
ALP (U/L)	45	272 ± 173	26	273 ± 149	19	271 ± 205
ChE (U/L)	35	368 ± 98	18	374 ± 114	17	361 ± 82
T-Bil (mg/dL)	44	0.55 ± 0.25	25	0.57 ± 0.26	19	0.52 ± 0.24
TP (g/dL)	47	7.77 ± 0.57	26	7.78 ± 0.51	21	7.74 ± 0.65
Alb (g/dL)	48	4.25 ± 0.72	25	4.32 ± 0.84	23	4.17 ± 0.59
UA (mg/dL)	46	5.39 ± 1.32	25	5.59 ± 1.24	21	5.15 ± 1.40
BUN (mg/dL)	48	16.4 ± 7.4	27	16.4 ± 8.0	21	16.3 ± 6.7
Cre (mg/dL)	50	0.77 ± 0.86	27	0.96 ± 1.13	23	0.54 ± 0.19
eGFRcre (mL/min/1.73 m^2^)	50	98.3 ± 36.3	27	92.3 ± 28.5	23	105.3 ± 43.3
BSA-uncorrected eGFRcre (mL/min)	50	77.8 ± 27.0	27	79.6 ± 24.8	23	75.5 ± 29.8
eGFRcys (mL/min/1.73 m^2^)	15	83.2 ± 29.5	10	86.1 ± 30.3	5	77.4 ± 30.3
Na (mEq/L)	48	140 ± 3	26	139 ± 3	22	140 ± 3
K (mEq/L)	48	4.27 ± 0.39	26	4.26 ± 0.43	22	4.27 ± 0.36
Cl (mEq/L)	47	104 ± 3	25	104 ± 3	22	104 ± 4
TC (mg/dL)	43	193 ± 32	25	196 ± 33	18	189 ± 31
TG (mg/dL)	49	161 ± 96	26	178 ± 98	23	143 ± 92
LDL-C (direct) (mg/dL)	39	119 ± 27	20	121 ± 28	19	117 ± 26
HDL-C (mg/dL)	46	57 ± 21	23	56 ± 20	23	59 ± 21
HbA1c (%)	46	6.42 ± 1.25	23	6.29 ± 0.98	23	6.55 ± 1.49
FPG (mg/dL)	21	118 ± 29	10	123 ± 36	11	112 ± 21
PPG (mg/dL)	24	142 ± 55	13	151 ± 47	11	132 ± 63

^a^*N* denotes the number of patients who had data. Regarding medications for diabetes, dyslipidemia, and hypertension, N means the patients with diabetes, dyslipidemia, and hypertension. Abbreviations: IGT: Impaired glucose tolerance; AP: Angina pectoris; MI: Myocardial infarction; PAD: peripheral artery disease; DPP-4: Dipeptidyl peptidase-4; alpha GI: alpha glucosidase inhibitor; SGLT2: Sodium-glucose cotransporter-2; GLP-1: Glucagon-like peptide-1; EPA: Eicosapentaenoic acid; Ca: Calcium; ARB: Angiotensin- II receptor blocker; ACE: Angiotensin-converting enzyme inhibitor; BMI: body mass index; SMI: skeletal muscle mass index; WBC: white blood cell; RBC: red blood cell; Hgb: hemoglobin; Plt: platelet; AST: aspartate aminotransferase; ALT: alanine aminotransferase; γ-GTP: gamma-glutamyl transpeptidase; LDH: lactate dehydrogenase; ALP: alkaline phosphatase; ChE: cholinesterase; T-Bil: total bilirubin; TP: total protein; Alb: albumin; UA: uric acid; BUN: blood urea nitrogen; Cre: creatinine; BSA: body surface are; eGFR: estimated glomerular filtration rate; Na: natrium; K: potassium; Cl: chlorine; TC: total cholesterol; TG: triglyceride; LDL-C: low-density lipoprotein cholesterol; HDL-C: high-density lipoprotein cholesterol; HbA1c: glycated hemoglobin; FPG: fasting plasma glucose; PPG: postprandial plasma glucose; SD: standard deviation.

Regarding comorbidities, the malignant neoplasm was found in about 25% of the patients. The specific types identified were as follows: breast cancer, thyroid cancer (follicular and papillary), colon cancer, bladder cancer, lung cancer, meningioma, malignant melanoma, undifferentiated polymorphic sarcoma, osteosarcoma, and soft tissue sarcoma.

Regarding the drugs, the most commonly used drug for diabetes mellitus was thiazolidine in 42.9% (15/35), followed by dipeptidyl peptidase-4 inhibitor (DPP4i) and metformin in 34.3% (12/35) of the patients with diabetes. Approximately 58.8% (20/34) of the patients with dyslipidemia were administered a statin. As for hypertension, calcium channel blockers followed by angiotensin II receptor blockers (ARBs) were the two most common types of drugs administered.

Regarding the blood examination, the gamma-glutamyl transpeptidase (γGTP) level was twice higher than the upper normal limit (for males 13-64, females 9-32 U/L). As for lipid profile, the mean low-density lipoprotein cholesterol (LDL-C) level was 121 ± 28 mg/dL for men and the mean triglyceride (TG) level was 161 ± 96 mg/dL, which were both slightly higher values than the normal range (for patients with diabetes, LDL-C 120 mg/dL, TG 150 mg/dL). Concerning the glucose profile, the mean glycated hemoglobin (HbA1c) was 6.42 ± 1.25%, the mean fasting plasma glucose was 118 ± 29 mg/dL, and the mean postprandial plasma glucose was 142 ± 55 mg/dL, which were all values within the target range (HbA1c 6.5%, fasting plasma glucose 126 mg/dL, postprandial plasma glucose 200 mg/dL). As for renal function, while the estimated glomerular filtration rate calculated from serum creatinine (eGFRcre) was 98.3 ± 36.3 mL/min/1.73 m^2^, the body surface area (BSA)-uncorrected eGFRcre was 77.8 ± 27.0 mL/min. The GFR calculated from cystatin C (eGFRcys) was 83.2 ± 29.5 mL/min/1.73 m^2^. Dissociation was observed between eGFRcre, BSA-uncorrected eGFRcre, and eGFRcys (*P* = 0.0017).

### Werner Syndrome Registry longitudinal analysis

Table 2 shows the comparison between the point of registration and one year later. Although there were no significant changes in physical examination findings compared to the baseline regarding major signs, the proportion of patients with intractable skin ulcers tended to increase (62.5% vs. 75.0%, *P* = 0.350). Regarding comorbidities, the percentage of patients with malignant neoplasms tended to increase (20.8% vs. 25.0%, *P* = 1.000); four patients died from malignant neoplasms (lung cancer and undifferentiated polymorphic sarcoma, osteosarcoma, soft tissue sarcoma, and malignant melanoma), and one patient died from renal failure.

**Table 2:** Comparison between the point of registration and one year later.

	***N***	**% (*n* patients with this characteristic)**		***P*-value**
		**at registration**	**one year later**	
**Major signs**				
Graying and/or loss of hair	24	100 (24)	100 (24)	−
Bilateral cataracts	24	100 (24)	100 (24)	−
Skin changes	24	95.8 (23)	95.8 (23)	1.000
Intractable skin ulcers	24	62.5 (15)	75.0 (18)	0.350
Soft tissue calcification	22	86.4 (19)	90.9 (20)	0.635
Bird-like face	24	83.3 (20)	87.5 (21)	0.683
High-pitched voice	24	83.3 (20)	83.3 (20)	1.000
**Clinical symptoms**				
Diabetes, IGT	24	70.8 (17)	79.2 (19)	0.505
Dyslipidemia	24	79.2 (19)	83.3 (20)	0.712
Hypertension	22	45.5 (10)	36.4 (8)	0.540
Fatty liver	22	54.5 (12)	50.0 (11)	0.763
AP or MI	24	4.2 (1)	8.3 (2)	0.551
PAD	24	4.2 (1)	4.2 (1)	1.000
Malignant neoplasm	24	20.8 (5)	25.0 (6)	1.000
**Medications**				
(1) For diabetes				
DPP-4 inhibitor	23	21.7 (5)	26.1 (6)	0.730
Biguanide	23	26.1 (6)	34.8 (8)	0.522
Thiazolidine	23	34.8 (8)	30.4 (7)	0.753
Alpha GI	24	8.3 (2)	4.2 (1)	0.551
Sulfonylurea	24	8.3 (2)	4.2 (1)	0.551
SGLT2 inhibitor	24	4.2 (1)	0 (0)	0.312
GLP-1 analog	24	4.2 (1)	8.3 (2)	0.551
Insulin	24	12.5 (3)	12.5 (3)	1.000
(2) For dyslipidemia				
Statin	23	21.7 (5)	26.1 (6)	0.730
Fibrate	23	26.1 (6)	34.8 (8)	0.522
Ezetimibe	23	34.8 (8)	30.4 (7)	0.753
EPA	24	8.3 (2)	4.2 (1)	0.551
Ion exchange resin	24	8.3 (2)	4.2 (1)	0.551
(3) For hypertension and others				
Ca blocker	24	20.8 (5)	20.8 (5)	1.000
ARB	24	12.5 (3)	8.3 (2)	0.637
Beta blocker	24	8.3 (2)	8.3 (2)	1.000
Diuretics	24	0 (0)	4.2 (1)	0.312
Aspirin	24	4.2 (1)	4.2 (1)	1.000
Antiplatelet	23	8.7 (2)	13.0 (3)	0.636
Anticoagulant	23	4.3 (1)	4.3 (1)	1.000
	***N***	**Mean ± SD**		***P*-value**
		**at registration**	**one year later**
**Physical findings/function**				
Body weight (kg)	21	45.1 ±9.7	44.9 ± 10.0	0.383
BMI (kg/m^2^)	21	18.4 ± 2.8	18.0 ± 2.9	0.279
Systolic blood pressure (mmHg)	19	124.0 ± 16.7	127.0± 18.0	0.628
Diastolic blood pressure (mmHg)	19	72.3 ± 9.5	73.7 ± 11.3	0.571
Pulse (/min)	13	87.1 ± 14.3	88.7 ± 13.4	0.570
Waist circumference (cm)	8	80.3 ± 15.6	79.4 ± 15.3	0.500
Mean grip strength (right) (kg)	8	18.6 ± 7.8	19.6 ± 7.9	0.469
Mean grip strength (left) (kg)	7	17.3 ± 7.4	17.4 ± 6.9	1.000
**Blood examinations**				
WBC (/μL)	19	7266 ± 1838	6300 ± 1988	0.003
RBC (x10^4^/μL)	19	455.2 ± 60.1	435.2 ± 75.4	0.447
Hb (g/dL)	19	13.42 ± 1.88	13.05 ± 2.27	0.574
Plt (x10^4^/μL)	19	28.3 ± 6.8	26.5 ± 9.9	0.050
AST (U/L)	20	30.3 ± 12.2	30.7 ± 13.4	0.958
ALT (U/L)	20	44.4 ± 29.2	37 ± 25.5	0.383
γGTP (U/L)	20	96.6 ± 141.9	61.3 ± 80.3	0.010
LDH (U/L)	18	195.9 ± 36.7	191.7 ± 39.6	0.827
ALP (U/L)	17	269.9 ± 197.8	207.9 ± 67.4	0.289
ChE (U/L)	16	395.1 ± 78.0	376.9 ± 71.6	0.323
T-Bil (mg/dL)	13	0.57 ± 0.14	0.43 ± 0.25	0.043
TP (g/dL)	19	8.02 ± 0.51	7.84 ± 0.49	0.172
Alb (g/dL)	18	4.54 ± 0.58	4.47 ± 0.48	0.510
UA (mg/dL)	18	5.37 ± 1.42	5.23 ± 1.36	0.641
BUN (mg/dL)	20	17.2 ± 7.5	16.5 ± 6.9	0.595
Cre (mg/dL)	20	0.775 ± 0.284	0.801 ± 0.293	0.157
eGFRcre (mL/min/1.73 m^2^)	20	85.4 ± 24.1	82.8 ± 27.6	0.408
BSA-uncorrected eGFRcre (mL/min)	17	70.8 ± 23.2	69.4 ± 26.6	0.109
Na (mEq/L)	20	139.7 ± 1.3	140 ± 1.7	0.486
K (mEq/L)	20	4.39 ± 0.31	4.37 ± 0.39	0.624
Cl (mEq/L)	20	104.5 ± 2.2	104.9 ± 2.7	0.123
TC (mg/dL)	16	187.4 ± 29.1	164.1 ± 26.2	0.021
TG (mg/dL)	19	172.3 ± 93.2	153 ± 74.4	0.390
LDL-C (direct) (mg/dL)	13	118.4 ± 28.5	97.5 ± 20.4	0.033
HDL-C (mg/dL)	18	54.0 ± 10.6	56.1 ± 13.1	0.424
HbA1c (%)	18	6.43 ± 0.84	6.08 ± 0.63	0.030
FPG (mg/dL)	5	115.4 ± 40.1	105.4 ± 10.5	0.625
PPG (mg/dL)	8	142.9 ± 29.9	152.4 ± 38.7	0.641

Abbreviations: IGT: Impaired glucose tolerance; AP: Angina pectoris; MI: Myocardial infarction; PAD: peripheral artery disease; DPP-4: Dipeptidyl peptidase-4; alpha GI: alpha glucosidase inhibitor; SGLT2: Sodium-glucose cotransporter-2; GLP-1: Glucagon-like peptide-1; EPA: Eicosapentaenoic acid; Ca: Calcium; ARB: Angiotensin- II receptor blocker; ACE: Angiotensin-converting enzyme inhibitor; BMI: body mass index; SMI: skeletal muscle mass index; WBC: white blood cell; RBC: red blood cell; Hgb: hemoglobin; Plt: platelet; AST: aspartate aminotransferase; ALT: alanine aminotransferase; γ-GTP: gamma-glutamyl transpeptidase; LDH: lactate dehydrogenase; ALP: alkaline phosphatase; ChE: cholinesterase; T-Bil: total bilirubin; TP: total protein; Alb: albumin; UA: uric acid; BUN: blood urea nitrogen; Cre: creatinine; BSA: body surface are; eGFR: estimated glomerular filtration rate; Na: natrium; K: potassium; Cl: chlorine; TC: total cholesterol; TG: triglyceride; LDL-C: low-density lipoprotein cholesterol; HDL-C: high-density lipoprotein cholesterol; HbA1c: glycated hemoglobin; FPG: fasting plasma glucose; PPG: postprandial plasma glucose; SD: standard deviation.

Blood examinations showed significant decreases in the white blood cell count (7266/μL vs. 6300/μL, *P* = 0.003), γGTP (96.6 mg/dL vs. 61.3 mg/dL, *P* = 0.010), and total bilirubin (T-Bil; 0.57 mg/dL vs. 0.43 mg/dL, *P* = 0.043). Significant decreases in total cholesterol (TC; 187.4 mg/dL vs. 164.1 mg/dL, *P* = 0.021), LDL-C (118.4 mg/dL vs. 97.5 mg/dL, *P* = 0.033), and HbA1c (6.43% vs. 6.08%, *P* = 0.030) were also observed.

Table 3 shows the comparison between the point of registration and two years later. Due to the difference in patients’ enrolled period, the number of observed patients decreased by the end of the study. The mean body weight decreased significantly (44.4 kg vs. 43.0 kg, *P* = 0.029). One patient died from brainstem hemorrhage caused by a myelodysplastic syndrome with overt acute myeloid leukemia infiltration to the central nervous system.

**Table 3:** Comparison between the point of registration and two years later.

	***N***	**% (*n* patients with this characteristic)**		***P*-value**
		**at registration**	**two years later**	
**Major signs**				
Graying and/or loss of hair	20	100 (20)	100 (20)	−
Bilateral cataracts	20	100 (20)	100 (20)	−
Skin changes	20	95 (19)	100 (20)	0.311
Intractable skin ulcers	20	60 (12)	75 (15)	0.311
Soft tissue calcification	20	85 (17)	85 (17)	1.000
Bird-like face	20	85 (17)	80 (16)	0.677
High-pitched voice	20	80 (16)	80 (16)	1.000
**Clinical symptoms**				
Diabetes, IGT	20	75 (15)	80 (16)	0.705
Dyslipidemia	20	75 (15)	80 (16)	0.705
Hypertension	19	42.1 (8)	42.1 (8)	1.000
Fatty liver	19	47.4 (9)	52.6 (10)	1.000
Cerebral bleeding	20	0 (0)	5 (1)	0.311
Cerebral infarction	20	0 (0)	5 (1)	0.311
AP or MI	20	5 (1)	5 (1)	1.000
PAD	20	0 (0)	5 (1)	0.311
Malignant neoplasm	20	25.0 (5)	35.0 (7)	0.731
**Medications**				
(1) For diabetes				
DPP-4 inhibitor	19	31.6 (6)	26.3 (5)	1.000
Biguanide	19	21.1 (4)	36..8 (7)	0.476
Thiazolidine	19	47.4 (9)	31.6 (6)	0.508
Alpha GI	19	5.3 (1)	0 (0)	1.000
Sulfonylurea	19	10.5 (2)	5.3 (1)	1.000
GLP-1 analog	19	5.3 (1)	10.5 (2)	1.000
Insulin	19	15.8 (3)	15.8 (3)	1.000
(2) For dyslipidemia				
Statin	19	63.2 (12)	52.6 (10)	0.743
Fibrate	19	5.3 (1)	10.5 (2)	1.000
EPA	19	5.3 (1)	5.3 (1)	1.000
Nicotinic acids	19	10.5 (2)	5.3 (1)	1.000
(3) For hypertension, and others				
Ca blocker	19	21.1 (4)	31.6 (6)	0.714
ARB	19	15.8 (3)	5.3 (1)	0.604
Beta blocker	19	10.5 (2)	10.5 (2)	1.000
Aspirin	19	5.3 (1)	5.3 (1)	1.000
Antiplatelet	18	5.6 (1)	5.6 (1)	1.000
Anticoagulant	18	5.6 (1)	0 (0)	1.000
	***N***	**Mean ± SD**		***P*-value**
		**at registration**	**two years later**	
**Physical findings/function**				
Body weight (kg)	19	44.4 ±9.5	43.0 ± 10.3	0.029
BMI (kg/m^2^)	18	18.0 ± 2.9	17.5 ± 3.3	0.056
Systolic blood pressure (mmHg)	15	123.9 ± 15.9	119.1± 17.3	0.482
Diastolic blood pressure (mmHg)	15	72.5 ± 10.4	70.3 ± 8.9	0.270
Pulse (/min)	6	84.7 ± 17.3	81.5 ± 17.2	0.719
Waist circumference (cm)	6	74.6 ± 11.0	74.9 ± 10.2	0.625
Mean grip strength (right) (kg)	5	16.5 ± 6.9	15.0 ± 4.1	0.438
Mean grip strength (left) (kg)	4	13.5 ± 6.5	12.1 ± 3.1	0.625
**Blood examinations**				
WBC (/μL)	16	6859 ± 1696	7186 ± 2261	0.836
RBC (x10^4^/μL)	16	442.6 ± 52.8	412.6 ± 89.3	0.298
Hb (g/dL)	16	13.05 ± 1.79	12.66 ± 2.85	0.850
Plt (x10^4^/μL)	16	28.33 ± 6.64	64.53 ± 106.3	0.744
AST (U/L)	17	26.4 ± 9.6	28.3 ± 9.9	0.551
ALT (U/L)	17	35.8 ± 27.4	31.6 ± 20.8	0.923
γGTP (U/L)	16	82.4 ± 129.3	71.1 ± 78.4	0.536
LDH (U/L)	14	195.9 ± 39.6	202.4 ± 46.6	0.366
ALP (U/L)	13	248.8 ± 215.0	205.0 ± 111.2	0.906
ChE (U/L)	12	374.7 ± 70.8	373.8 ± 90.5	0.733
T-Bil (mg/dL)	9	0.55 ± 0.16	0.34 ± 0.29	0.039
TP (g/dL)	16	7.96 ± 0.54	7.78 ± 0.61	0.774
Alb (g/dL)	15	4.44 ± 0.59	4.27 ± 0.68	0.668
UA (mg/dL)	15	5.38 ± 1.45	4.72 ± 0.88	0.089
BUN (mg/dL)	16	17.7 ± 8.0	17.2 ± 6.7	0.657
Cre (mg/dL)	17	0.766 ± 0.311	0.788 ± 0.386	0.451
eGFRcre (mL/min/1.73 m^2^)	17	84.5 ± 25.6	87.6 ± 41.6	0.644
BSA-uncorrected eGFRcre (mL/min)	15	68.5 ± 22.8	70.6 ± 33.6	0.639
Na (mEq/L)	16	139.7 ± 1.4	138.8 ± 3.6	0.461
K (mEq/L)	16	4.33 ± 0.33	5.48 ± 3.65	0.022
Cl (mEq/L)	16	104.7 ± 2.2	97.9 ± 26.2	0.892
TC (mg/dL)	12	182.0 ± 23.6	173.6 ± 26.5	0.531
TG (mg/dL)	15	146.1 ± 79.1	141.2 ± 53.4	0.836
LDL-C (direct) (mg/dL)	11	113.2 ± 27.9	101.4 ± 27.3	0.182
HDL-C (mg/dL)	14	57.1 ± 10.7	55.2 ± 11.1	0.726
HbA1c (%)	16	6.26 ± 0.65	6.31 ± 1.02	0.550
PPG (mg/dL)	7	137.0 ± 26.9	148.1 ± 34.3	0.375

Abbreviations: IGT: Impaired glucose tolerance; AP: Angina pectoris; MI: Myocardial infarction; PAD: peripheral artery disease; DPP-4: Dipeptidyl peptidase-4; alpha GI: alpha glucosidase inhibitor; SGLT2: Sodium-glucose cotransporter-2; GLP-1: Glucagon-like peptide-1; EPA: Eicosapentaenoic acid; Ca: Calcium; ARB: Angiotensin- II receptor blocker; ACE: Angiotensin-converting enzyme inhibitor; BMI: body mass index; SMI: skeletal muscle mass index; WBC: white blood cell; RBC: red blood cell; Hgb, hemoglobin; Plt: platelet; AST, aspartate aminotransferase; ALT: alanine aminotransferase; γ-GTP: gamma-glutamyl transpeptidase; LDH: lactate dehydrogenase; ALP: alkaline phosphatase; ChE: cholinesterase; T-Bil: total bilirubin; TP: total protein; Alb: albumin; UA: uric acid; BUN: blood urea nitrogen; Cre: creatinine; BSA: body surface are; eGFR: estimated glomerular filtration rate; Na: natrium; K: potassium; Cl: chlorine; TC: total cholesterol; TG: triglyceride; LDL-C: low-density lipoprotein cholesterol; HDL-C: high-density lipoprotein cholesterol; HbA1c: glycated hemoglobin; FPG: fasting plasma glucose; PPG: postprandial plasma glucose; SD: standard deviation.

The blood examination showed that glucose and lipid metabolism were controlled within the target range, and T-Bil decreased (0.55 mg/dL vs. 0.34 mg/dL, *P* = 0.039), while a new increase in the mean serum potassium was observed (4.33 mEq/L vs. 5.48 mEq/L, *P* = 0.022). Regarding therapeutic drugs, metformin tended to increase, while thiazolidine tended to decrease in association with diabetes, and the use of ARBs as antihypertensive drugs were discontinued in two patients using them.

Table 4 shows the comparison between the point of registration and three years later. A significant decrease in renal function was observed over time. At the point of registration, the mean eGFRcre was 74.8 mL/min/1.73/m^2^, and the mean BSA-uncorrected eGFRcre was 59.3 mL/min, whereas three years later, the mean eGFRcre was 63.4 mL/min/1.73 m^2^ (*P* = 0.078) and the mean BSA-uncorrected eGFRcre was 50.2 mL/min (*P* = 0.047), which correlated to a decrease of approximately 3 mL/min (/1.73 m^2^) per a year on average.

**Table 4:** Comparison between the point of registration and three years later.

	***N***	**% (*n* patients with this characteristic)**		***P*-value**
		**at registration**	**three years later**	
**Major signs**				
Graying and/or loss of hair	9	100 (9)	100 (9)	−
Bilateral cataracts	9	100 (9)	100 (9)	−
Skin changes	9	88.9 (8)	100 (9)	1.000
Intractable skin ulcers	9	66.7 (6)	77.8 (7)	1.000
Soft tissue calcification	9	100 (9)	100 (9)	-
Bird-like face	9	77.8 (7)	77.8 (7)	1.000
High-pitched voice	9	77.8 (7)	88.9 (8)	1.000
**Clinical symptoms**				
Diabetes, IGT	9	55.6 (5)	66.7 (6)	1.000
Dyslipidemia	9	66.7 (6)	77.8 (7)	1.000
Hypertension	8	50.0 (4)	62.5 (5)	1.000
Fatty liver	9	55.6 (5)	66.7 (6)	1.000
PAD	9	0 (0)	11.1 (1)	1.000
Malignant neoplasm	9	44.4 (4)	44.4 (4)	1.000
**Medications**				
(1) For diabetes				
DPP-4 inhibitor	9	22.2 (2)	33.3 (3)	1.000
Biguanide	9	11.1 (1)	22.2 (2)	1.000
Thiazolidine	9	22.2 (2)	11.1 (1)	1.000
GLP-1 analog	9	11.1 (1)	11.1 (1)	1.000
Insulin	9	11.1 (1)	11.1 (1)	1.000
(2) For dyslipidemia				
Statin	9	55.6 (5)	55.6 (5)	1.000
Fibrate	9	11.1 (1)	11.1 (1)	1.000
EPA	9	11.1 (1)	11.1 (1)	1.000
Nicotinic acids	9	22.2 (2)	11.1 (1)	1.000
(3) For hypertension and others				
Ca blocker	8	37.5 (3)	50.0 (4)	1.000
ARB	8	12.5 (1)	0 (0)	1.000
Beta blocker	8	12.5 (1)	12.5 (1)	1.000
Antiplatelet	8	12.5 (1)	12.5 (1)	1.000
Anticoagulant	8	12.5 (1)	0 (0)	1.000
	***N***	**Mean ± SD**		***P*-value**
		**at registration**	**three years later**	
**Physical findings/function**				
Body weight (kg)	7	45.29 ±4.62	44.30 ± 4.57	0.281
BMI (kg/m^2^)	3	18.06 ± 1.43	17.35 ± 1.69	0.500
Systolic blood pressure (mmHg)	5	132.8 ± 21.6	138.4± 15.8	0.625
Diastolic blood pressure (mmHg)	5	74.0 ± 10.77	76.6 ± 12.54	0.875
Pulse (/min)	4	92.5 ± 12.0	93.5 ± 22.2	0.875
**Blood examinations**				
WBC (/μL)	6	7517 ± 1699	8550 ± 2671	0.313
RBC (x10^4^/μL)	6	424.8 ± 69.7	394.7 ± 63.2	0.156
Hb (g/dL)	6	12.05 ± 2.02	12.15 ± 1.82	0.688
Plt (x10^4^/μL)	6	29.23 ± 5.44	33.27 ± 7.01	0.156
AST (U/L)	7	24.7 ± 4.2	24.3 ± 9.6	1.000
ALT (U/L)	8	28.6 ± 14.5	27.8 ± 17.4	0.734
γGTP (U/L)	8	43.5 ± 32.3	58.4 ± 55.9	0.398
LDH (U/L)	7	195.9 ± 39.9	194.9 ± 26.5	0.563
ALP (U/L)	6	204.5 ± 100.9	241.2 ± 69.4	0.156
ChE (U/L)	6	374.2 ± 98.0	376.0 ± 102.2	1.000
TP (g/dL)	7	7.94 ± 0.20	8.14 ± 0.59	0.641
Alb (g/dL)	7	4.34 ± 0.69	4.36 ± 0.29	1.000
UA (mg/dL)	8	5.56 ± 1.40	4.91 ± 1.03	0.109
BUN (mg/dL)	7	23.1 ± 9.1	25.6 ± 12.3	0.438
Cre (mg/dL)	7	0.904 ± 0.425	1.133 ± 0.747	0.078
eGFRcre (mL/min/1.73 m^2^)	7	74.8 ± 33.5	63.4 ± 31.9	0.078
BSA-uncorrected eGFRcre (mL/min)	7	59.3 ± 25.2	50.2 ± 26.9	0.047
Na (mEq/L)	7	139.3 ± 1.6	139.7 ± 2.0	0.531
K (mEq/L)	7	4.51 ± 0.37	4.96 ± 0.82	0.141
Cl (mEq/L)	7	104.6 ± 2.9	105.0 ± 3.8	0.922
TC (mg/dL)	6	179.8 ± 16.7	189.7 ± 25.9	0.563
TG (mg/dL)	7	114.1 ± 25.8	126.6 ± 55.6	0.578
LDL-C (direct) (mg/dL)	4	115.0 ± 17.9	103.3 ± 11.9	0.375
HDL-C (mg/dL)	6	52.3 ± 8.8	54.2 ± 8.9	0.375
HbA1c (%)	7	6.20 ± 0.70	6.17 ± 0.91	0.875
PPG (mg/dL)	3	144.0 ± 24.8	139.0 ± 11.1	1.000

Abbreviations: IGT: Impaired glucose tolerance; AP: Angina pectoris; MI: Myocardial infarction; PAD: peripheral artery disease; DPP-4: Dipeptidyl peptidase-4; alpha GI: alpha glucosidase inhibitor; SGLT2: Sodium-glucose cotransporter-2; GLP-1: Glucagon-like peptide-1; EPA: Eicosapentaenoic acid; Ca: Calcium; ARB: Angiotensin- II receptor blocker; ACE: Angiotensin-converting enzyme inhibitor; BMI: body mass index; SMI: skeletal muscle mass index; WBC: white blood cell; RBC: red blood cell; Hgb: hemoglobin; Plt: platelet; AST: aspartate aminotransferase; ALT: alanine aminotransferase; γ-GTP: gamma-glutamyl transpeptidase; LDH: lactate dehydrogenase; ALP: alkaline phosphatase; ChE: cholinesterase; T-Bil: total bilirubin; TP: total protein; Alb: albumin; UA: uric acid; BUN: blood urea nitrogen; Cre: creatinine; BSA: body surface are; eGFR: estimated glomerular filtration rate; Na: natrium; K: potassium; Cl: chlorine; TC: total cholesterol; TG: triglyceride; LDL-C: low-density lipoprotein cholesterol; HDL-C: high-density lipoprotein cholesterol; HbA1c: glycated hemoglobin; FPG: fasting plasma glucose; PPG: postprandial plasma glucose; SD: standard deviation.

The results of the survey covering the entire period regarding malignant neoplasms, renal function, and aspiration pneumonia are described below.

Regarding malignant neoplasms, the morbidity due to malignant neoplasms at baseline was 25.5% (out of 51 patients) and 31.4% in the entire survey period. Table 5 shows the type of malignant neoplasms and the age of onset. The breakdown of the malignant neoplasm types revealed epithelial neoplasms in eight patients (15.7%), non-epithelial in four patients (7.8%), and both in three patients (5.9%). Multiple neoplasms were found in 3/51 (5.9%) of all the patients enrolled, and 3/16 (18.8%) of the patients with malignant neoplasms.

**Table 5:** Type of malignant neoplasms and age of onset.

**Patient No.**	**Age of onset (years)**	**Type of malignant neoplasm**	**Age during reporting or die (years)**	**Status**
1	32	Bladder cancer	61	Alive
2	36	Breast cancer	44	Alive
3	42	Colon cancer	56	Alive
4	48, 49	Papillary thyroid cancer, meningioma^a^	49	Alive
5	48	Myelodysplastic syndrome^a^, acute myeloid leukemia^a^	48	Dead
6	50	Papillary thyroid cancer	50	Alive
7	52	Lung adenocarcinoma	53	Alive
8	52	Breast cancer	55	Alive
9	56	Melanoma^a^	58	Dead
10	57, 60, 64	Meningioma^a^, breast cancer, lung cancer	65	Alive
11	Unknown	Osteosarcoma^a^	45	Dead
12	Unknown	Thyroid follicular carcinoma	53	Alive
13	Unknown, unknown	Lung cancer, undifferentiated pleomorphic sarcoma^a^	55	Dead
14	Unknown	Soft tissue sarcoma^a^	56	Dead
15	Unknown	Lung adenocarcinoma	64	Alive
16	Unknown	Unknown	64	Dead

^a^Shows non-epithelial neoplasm.

**Figure 1:**
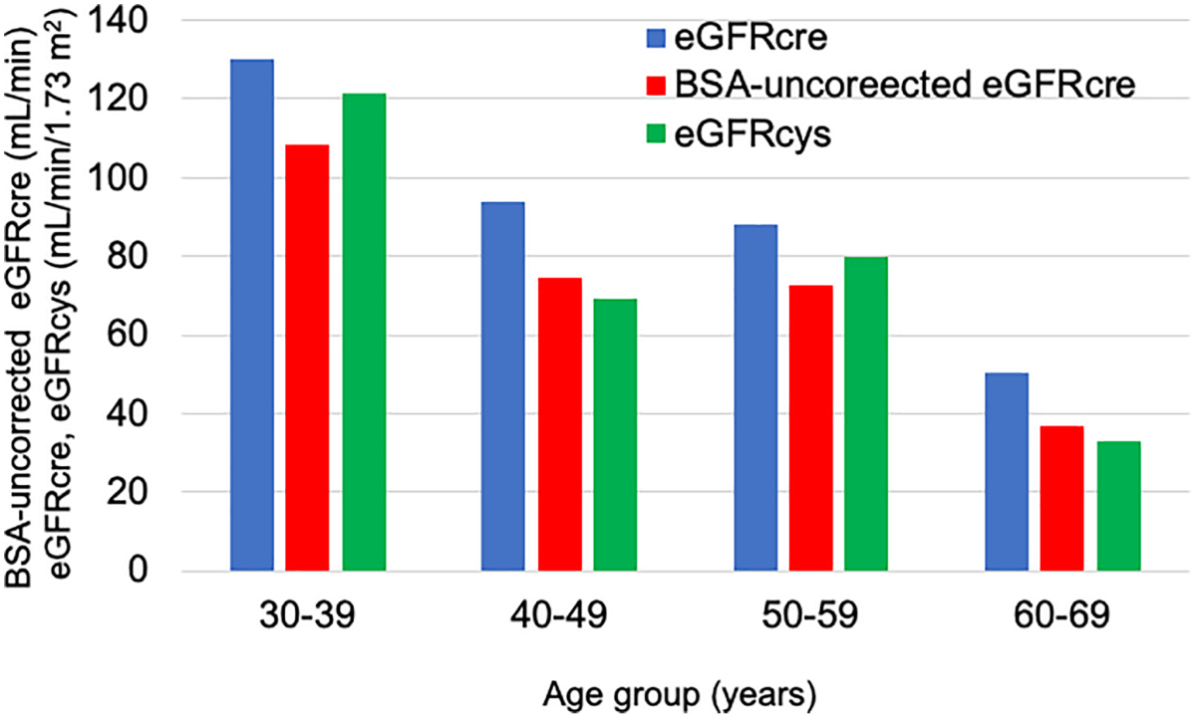
**Average renal function in each age group over the entire survey period.** The blue bar shows the eGFRcre. The red bar shows the BSA-uncorrected eGFRcre. The green bar shows the eGFRcys.

Regarding renal function, Figure 1 shows the age group and the mean renal function during the entire survey period. Of the three indices of renal function, there was a discrepancy between eGFRcre and BSA-uncorrected eGFRcre/eGFRcys. The mean eGFRcre, BSA-uncorrected eGFRcre, and eGFRcys for each age decile were as follows: 30s, 129.9/108.5/121.6; 40s, 93.7/74.5/69.0; 50s, 88.2/72.8/79.8; and 60s, 50.2/36.9/33.1.

Many patients with Werner syndrome have painful leg ulcers and frequently use non-steroidal anti-inflammatory drugs (NSAIDs). Frequent use of NSAIDs is connected with an observed decrease in renal function. Table 6 shows that in the relationship between the presence of leg ulcers and renal function, renal function tended to be low in patients with leg ulcers, and eGFRcre, BSA-uncorrected eGFRcre, and eGFRcys were as follows: patients with leg ulcers, 91.5 ± 30.5 mL/min/1.73 m^2^ vs. without leg ulcers, 112.9 ± 43.9 mL/min/1.73 m^2^, *P* = 0.121; 73.7 ± 25.2 mL/min vs. 86.3 ± 29.4 mL/min, *P* = 0.228; and 77.0 ± 27.5 mL/min/1.73 m^2^ vs. 100.1 ± 32.2 mL/min/1.73 m^2^, *P* = 0.240, respectively. In relation to the usage of NSAIDs, there was no difference in renal function between the users and non-users, as follows: NSAIDs user 94.0 ± 28.7 mL/min/1.73 m^2^ vs. non-user 99.2 ± 38.0 mL/min/1.73 m^2^, *P* = 0.830; 74.5 ± 26.3 mL/min vs. 78.5 ± 27.4 mL/min, *P* = 0.910, and 75.0 ± 9.2 mL/min/1.73 m^2^ vs. 85.2 ± 32.7 mL/min/1.73 m^2^, *P* = 0.471, respectively.

**Table 6:** Relationship between renal function and the presence of leg ulcers/the usage of NSAIDs.

	***N***	**Patients with ulcer (mean ± SD)**	***N***	**Patients without ulcer (mean ± SD)**	***P*-value**
eGFR (mL/min/1.73 m^2^)	34	91.5 ± 30.5	16	112.9 ± 43.9	0.121
BSA-uncorrected eGFRcre (mL/min)	34	73.7 ± 25.2	16	86.3 ± 29.4	0.228
eGFRcys (mL/min/1.73 m^2^)	11	77.0 ± 27.5	4	100.1 ± 32.2	0.240
	***N***	**Patients with NSAIDs (mean ± SD)**	***N***	**Patients without NSAIDs (mean ± SD)**	***P*-value**
eGFR (mL/min/1.73 m^2^)	9	94.0 ± 28.7	41	99.2 ± 38.0	0.830
BSA-uncorrected eGFRcre (mL/min)	9	74.5 ± 26.3	41	78.5 ± 27.4	0.910
eGFRcys (mL/min/1.73 m^2^)	3	75.0 ± 9.2	12	85.2 ± 32.7	0.471

Abbreviations: NSAIDs: non-steroidal anti-inflammatory drugs; eGFR: estimated glomerular filtration rate; BSA: body surface area.

Two patients (4.9%) were hospitalized with aspiration pneumonia; the age at onset was 67 years and 63 years, respectively.

During the four-year follow-up, deaths could be confirmed in six patients, of whom five died from malignant neoplasms and one from renal failure. The average age of death was 54.2 years (52.4 years for malignant neoplasms and 63.0 years for renal failure).

## DISCUSSION

Data from the registry described in this study revealed the occurrence of changes over time for up to four years after registration in patients with Werner syndrome in Japan. Notably, a novel finding was that the index of renal function may greatly deviate from the actual state of renal function and that the rate of decline in renal function may be rapid. Although there were case reports regarding declining renal function in a patient with Werner syndrome [12–15], there was no report with a large sample size and long-term follow-up. Moreover, it was clarified that the morbidity due to malignant neoplasms is higher than that of the general population, the age of onset is younger, and both epithelial and non-epithelial neoplasms exist in similar ratios. In addition, aspiration pneumonia was observed in patients in their sixties.

The life expectancy of patients with Werner syndrome is extending [7] due to improvements in understanding the pathology of Werner syndrome and the development of medical treatments for diabetes and dyslipidemia [9]. Therefore, longer-term, more detailed follow-ups and medical interventions are needed. Atherosclerotic cardiovascular diseases (ASCVD), malignant neoplasms, sarcopenia, and leg ulcers were mentioned as the main pathological conditions affecting the prognosis of patients with Werner syndrome. The medical intervention seems to be effective for improving life quality [16–18]. Each important comorbidity is now discussed as follows.

### Malignant neoplasms

In this study, the morbidity due to malignant neoplasms at baseline was 25.5% (13/51 patients). The morbidity due to malignant neoplasms in patients with Werner syndrome is increasing, and aging is a possible contributing factor [19]. In other words, patients with Werner syndrome are no longer dying from cardiovascular diseases at a young age; therefore, the morbidity due to malignant neoplasms may be increasing as a result.

Moreover, the ratio of epithelial neoplasms to non-epithelial neoplasms is generally approximately 10:1, while patients with Werner syndrome have very high morbidity associated with non-epithelial neoplasms [19, 20]. Similarly, the ratio of epithelial neoplasms to non-epithelial neoplasms was 12:7 for the entire study period.

Although the prevalence of epithelial neoplasms has been reported to be significantly higher in patients with diabetes compared to patients without diabetes [19], Lauper et al. reported no association between diabetes and epithelial or non-epithelial neoplasms [21]. In our study, the prevalence of epithelial neoplasms in patients with diabetes was 18.9%, and that in patients without diabetes was 28.6%.

**Figure 2:**
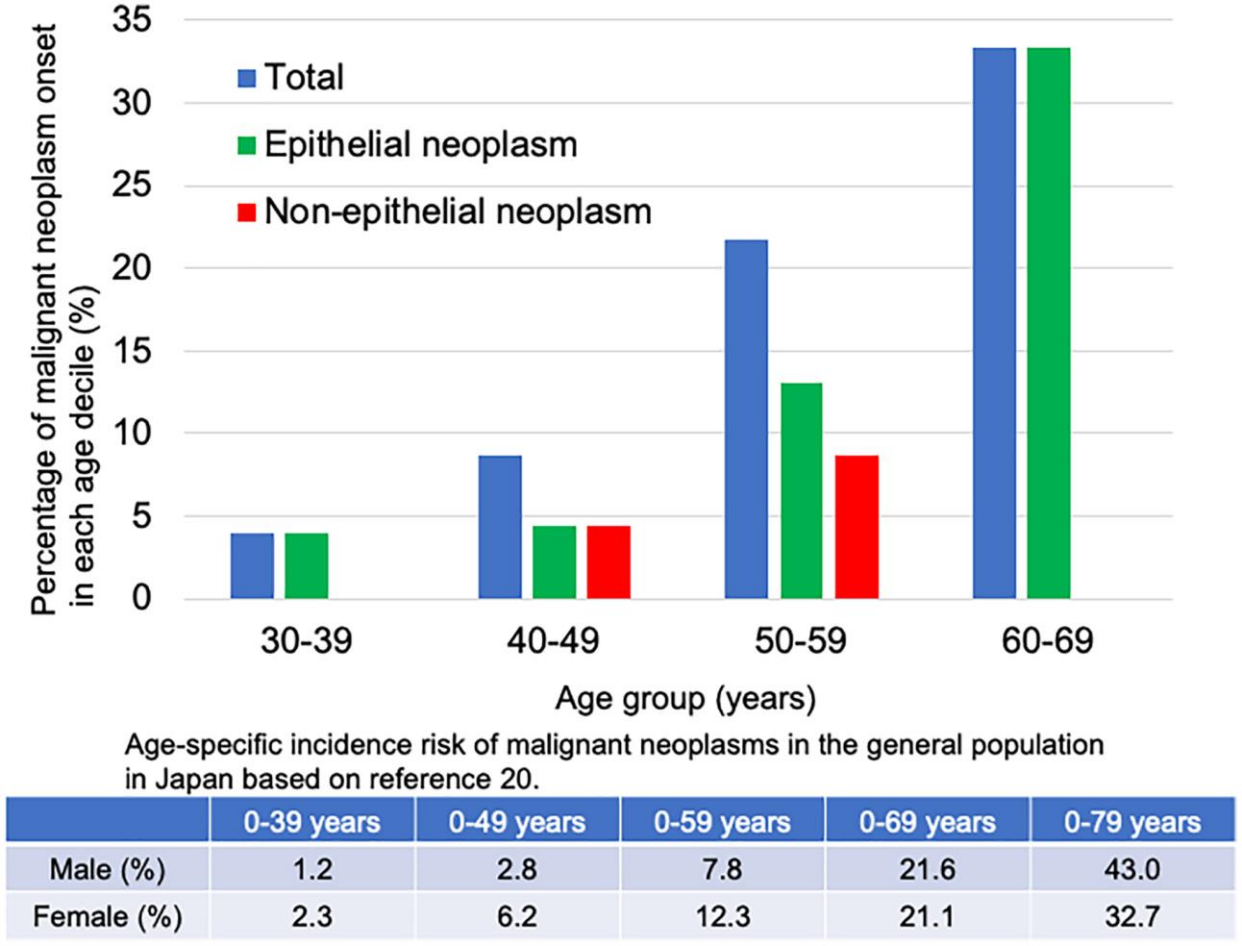
**Percentage of malignant neoplasms onset in each age decile in the patients with Werner syndrome.** The blue bar shows the percentage of total malignant neoplasms in each age group. The green bar shows the percentage of epithelial neoplasms in each age group. The red bar shows the percentage of non-epithelial neoplasms in each age group. Patients with thyroid follicular cancer, osteosarcoma, lung cancer/undifferentiated polymorphic sarcoma, and soft tissue sarcoma were excluded because the exact age of onset was unknown.

Furthermore, reportedly, the age of onset for malignant neoplasm was low in patients with Werner syndrome, with the predominant onset age for malignant neoplasms being 25–65 years. In particular, the morbidity due to non-epithelial neoplasms, in other words, those of mesenchymal origin (sarcoma), is particularly high (20%) in patients with Werner syndrome before the age of 41 years [22]. In other reports, the average age of malignant neoplasm onset was 47.2 years for epithelial neoplasms, 45.2 years for non-epithelial neoplasms, and 45.8 years for all malignant neoplasms [19]. Figure 2 shows the percentage of malignant neoplasms by onset in each age group. Epithelial neoplasms developed at a younger age, and the incidence of non-epithelial neoplasms increased during the late 40s. The average age of onset was 48.4 years for epithelial neoplasms, 52.5 years for non-epithelial neoplasms, and 49.7 years for all malignant neoplasms. Therefore, the age of onset of non-epithelial neoplasms is higher than in previous reports as well. This may be due to the fact that the patients’ lifespan has been prolonged by cardiovascular disease prevention and epithelial neoplasm treatment.

The table in Figure 2 shows the age-specific incidence risk of malignant neoplasms in the general population in Japan. Since the probability of malignant neoplasms in the 30s and 60s of the general population is 1.2–21.6%, the morbidity due to malignant neoplasms is higher in the same age group of patients with Werner syndrome. Malignant neoplasms onset is approximately 10 years earlier in patients with Werner syndrome than in the general population [23].

A high percentage of multiple neoplasms is also characteristic of patients with Werner syndrome. In other reports, there were multiple neoplasms in 5.3% of all patients and 15–20% of patients with malignant neoplasms [19, 24]. A similar tendency was observed in this study.

Reportedly, two-thirds of neoplasms in the Werner syndrome population were thyroid neoplasms, malignant melanomas, meningiomas, soft tissue sarcomas, leukemia and preleukemic conditions, and osteosarcoma and bone neoplasms; in our study, half of the patients with malignant neoplasms had these neoplasms, and the incidence of breast and lung cancers in the epithelial neoplasm group was also high [25]. Regarding osteosarcoma, X-ray screening of long bones may be useful. Therefore, screening of these neoplasms should be prioritized.

Currently, the main cause of death in patients with Werner syndrome is malignant neoplasms development, and malignant neoplasms greatly influence prognosis; therefore, early detection and treatment by regular screening from a younger age are both very important to ameliorate their prognosis.

There are some cautions to be considered regarding cancer treatment for patients with Werner syndrome. Reportedly, a patient with a heterozygous mutation in the WRN gene and a retroperitoneal liposarcoma had dramatic renal and hematological toxicity after cytotoxic chemotherapy [26]. For patients with a WRN mutation, close monitoring of the hematologic profile and renal function is needed to avoid severe toxicities. Furthermore, it was reported that radiotherapy is contraindicated in most homozygous patients with recessive radiosensitivity syndromes, including Werner syndrome [27]. Therefore, safer and more effective new cancer treatments for patients with Werner syndrome are needed.

Recently, the mechanism of high morbidity and treatment strategies for malignant neoplasm in patients with Werner syndrome have been getting clear. The Werner syndrome ATP-dependent helicase (WRN) is a RecQ enzyme involved in the maintenance of genome integrity. WRN is associated with Werner syndrome and a predisposition to multiple cancers, such as multiple myeloma [28], myelodysplastic syndrome/acute myeloid leukemia [29], colorectal cancer [30], breast cancer [31], and ovarian cancer [32]. WRN contributes to chromosomal stability for survival in both normal and cancer cells [33]. WRN is also required for DNA damage repair and the survival of cancer cells with microsatellite instability (MSI) [34]. WRN depletion induces double-strand DNA breaks and promotes apoptosis and cell cycle arrest selectively in MSI models. Loss of WRN leads to synthetic lethality in mismatch repair-deficient/high MSI cells [35].

Genome-wide screening studies have reported that WRN inhibition induces massive chromosome disruption [36] and synthetic lethality in cancer cells with high MSI [37, 38]. The development possibility of novel therapeutic agents that target WRN for MSI-associated cancers by pharmacological inhibition of WRN helicase function has been reported [35, 36, 38]. Reportedly, exposure to small-molecule compounds, such as NSC 19630 (N-(1-(4-((4-methoxyphenyl) amino) quinazolin-6-yl) ethyl)-3-(pyridin-3-yl) acrylamide) and NSC 617145 (5-(4-chlorophenyl)-4-(4-methylphenyl)-4,5-dihydro-1H-imidazol-2-amine N-(4-methylphenyl) sulfonylacetamide), which inhibits WRN helicase activity, sensitizes cancer cells to DNA-damaging agents [39] and DNA cross-linking agents [40]. Sublethal dosage of small-molecule compounds and the chemotherapy drug act synergistically to inhibit cell proliferation and induce DNA damage in cancer cells. These studies are still in progress and thus the results are highly awaited.

### Renal function

According to the renal function index, the discrepancy was observed in patients with Werner syndrome. It may be because patients with Werner syndrome have a smaller body size and less muscle mass than those without Werner syndrome.

The calculation of eGFRcre (mL/min/1.73 m^2^) requires the serum creatinine level, age, and sex. The calculation of BSA-uncorrected eGFRcre (mL/min) requires five items: serum creatinine level, age, sex, height, and weight. In other words, it considers the physique. The calculation of eGFRcys (mL/min/1.73 m^2^) requires three items: serum cystatin C level, age, and sex. Therefore, it is not affected by muscle mass.

Since creatinine is easily affected by muscle mass, diet, and exercise, eGFRcre is often higher in older adults with less muscle mass, and discrepancies are likely to be observed. The situation is similar to that in patients with Werner syndrome. Therefore, in the patients with Werner syndrome, it is considered that eGFRcre dissociates from BSA-uncorrected eGFRcre and eGFRcys.

It can be observed from Figure 1 that the eGFRcys/eGFRcre ratios are <1.0. Reportedly, the presence of low eGFRcys/eGFRcre ratios (<1.0) is associated with sarcopenia [41]. In Figure 1, the eGFRcys/eGFRcre ratio is also low in non-elderly people, which may reflect that patients with Werner syndrome are small and have low muscle mass even at a young age. Therefore, there are concerns due to the dissociation of the renal function indices. Side effects may likely occur when using medicines such as antibiotics and NSAIDs for patients with poor renal function. For patients with extremely small body size and decreased renal function, the use of BSA-uncorrected eGFRcre or eGFRcys is preferable to eGFRcre. It is important to carefully consider which renal function index to use before deciding the amount of medicine to be used.

The other problem is that when there is a worse eGFRcys than eGFRcre, it is associated with a higher risk of death and end-stage renal disease [42, 43]. Therefore, it is necessary to carefully follow the course of a patient’s renal function.

Renal function (eGFRcre, BSA-uncorrected eGFRcre) tended to decrease over three years, and BSA-uncorrected eGFRcre showed a significant decrease. The average rate of decline in BSA-uncorrected eGFRcre in the patients with Werner syndrome was 9.1 mL/min for three years, that is, approximately 3 mL/min in a year. In addition, as Figure 1 shows, the decrease in renal function is remarkable in the 30s and after the 50s. The average rate of decline in eGFR for general Japanese people aged 40 and above is 0.36 mL/min/1.73 m^2^ (males 40–49 years: 0.35 mL/min/1.73 m^2^; 50–59 years: 0.31 mL/min/1.73 m^2^; 60–69 years: 0.37 mL/min/1.73 m^2^; 70–79 years: 0.42 mL/min/1.73 m^2^; females 40–49 years: 0.41 mL/min/1.73 m^2^; 50–59 years: 0.31 mL/min/1.73 m^2^; 60–69 years: 0.32 mL/min/1.73 m^2^; and 70–79 years: 0.39 mL/min/1.73 m^2^) [44]. Therefore, this suggests that the rate of renal function decline was faster than that of the general population of the same age.

While the management of diabetes and hypertension was appropriate, the use of analgesics such as NSAIDs was considered one of the reasons that the rate of renal function decline was rapid. Approximately 70% of patients with Werner syndrome develop painful intractable ulcers, and NSAIDs are frequently used. Although there was no relationship between leg ulcers or NSAIDs and renal function in this three-year follow-up, various factors, such as the presence of peripheral angiopathy, also affect the results; therefore, it may be necessary to pay attention to the dose of NSAIDs in patients with painful intractable ulcers. The use of NSAIDs in older adults is often noted, and similar caution is needed in patients with Werner syndrome, even the non-elderly, whose renal dysfunction cannot be predicted by eGFRcre alone. For other medicines, such as antibiotics, anti-cancer drugs, and ARBs, it is also necessary to care about drug-induced renal damage or side effects due to overdose.

### Atherosclerotic cardiovascular diseases and metabolic diseases

ASCVDs, such as myocardial infarction, angina pectoris, and atherothrombotic cerebral infarction, were not common during the study period. The reduction of ASCVD appears to be extending the life expectancy of patients with Werner syndrome. This is presumed to be associated with the improved management of metabolic diseases such as diabetes, dyslipidemia, and hypertension. Regarding diabetes treatments, similar to findings in a previous report [9], pioglitazone, metformin, and insulin sensitizers were often used. While, longitudinally, the percentage of metformin use has increased more than that of pioglitazone, suggesting that the negative effects of pioglitazone on osteoporosis may be considered. Among those with dyslipidemia, statins were used in 58.8% of patients. Although the baseline data were generally within the control target range over four years, the LDL-C level decreased more from baseline, suggesting suitable interventions took place. Hypertriglyceridemia should also be noted. It was reported that a 29-year-old patient with Werner syndrome and hypertriglyceridemia (triglyceride level of 3900 mg/dL) had advanced three-vessel disease requiring coronary artery bypass graft surgery [45]. Therefore, in addition to statins, management of hypertriglyceridemia may also be needed. Regarding antihypertensive medications, the use of ARBs decreased in patients over the four years, and eventually, there were no patients using ARBs. Hyperkalemia was cited as one of the reasons for medication discontinuation, suggesting a relationship with impaired renal function. Although metabolic diseases were sufficiently controlled overall, as life expectancy prolongs, appropriate medications must be selected to address potential side effects. Moreover, patients with severe aortic stenosis [46, 47] and heart failure due to impaired coronary microcirculation with no coronary artery stenosis have been reported [48]. Therefore, a comprehensive evaluation of arteriosclerosis in patients with Werner syndrome is still needed.

### Sarcopenia

The sarcopenia diagnostic criteria by the Asian Working Group for Sarcopenia 2019 [49] has three indicators, of them, the following three indicators were used in this survey: 1) grip strength (male/female) <28 kg/<18 kg, 2) walking speed <1.0 m/s, and 3) SMI dual-energy X-ray absorptiometry <7.0 kg/m^2^ (male) and <5.4 kg/m^2^ (female), SMI bioimpedance, <7.0 kg/m^2^ (male) and <5.7 kg/m^2^ (female). As Table 1 shows, the mean values of grip strength and SMI in the present registry met the criteria of 1) and 3), and sarcopenia was suspected. The patients with Werner syndrome tended to have sarcopenia obesity (visceral fat increase despite low BMI). It was reported that sarcopenia tended to appear before visceral fat accumulation [50]. Moreover, over the years, body weight, BMI, and grip strength tended to decrease in this study. The adipogenic and chondrogenic differentiation capacity was significantly decreased in the foot fibroblasts of patients when Werner syndrome *in vitro*. It may partially explain the underlying mechanism of sarcopenia in patients with Werner syndrome [51]. The presence of sarcopenia may be related not only to QOL reduction but also to renal function evaluation, as aforementioned, suggesting that sarcopenia prevention is needed. Measures such as leucine intake [52, 53] and strength training [54, 55], which have general preventive effects on sarcopenia, should be considered. Ghrelin receptor agonist has been shown to increase body weight, muscle mass, and appetite in patients with cancer cachexia [56, 57]. These findings suggest that ghrelin receptor agonist also may be effective in patients with Werner syndrome, in whom skeletal muscle mass is significantly reduced.

### Other complications associated with aging

As lifespans prolong, complications such as aspiration pneumonia, which are often seen in late older adults, are increasing. Two patients with aspiration pneumonia were reported in this survey. They were aged in their 60s and were thus younger than the average age group that generally experienced aspiration pneumonia. Vaccinations to prevent pneumonia in those aged in their early 60s and oral care may be useful preventive measures. Further, the use of benzodiazepine should be avoided.

This study is limited because the evaluated registry does not cover all patients with Werner syndrome in Japan. However, almost half of the patients confirmed in a previous national survey registered; therefore, the clinical evidence was established based on a highly universal (and the largest) database of Werner syndrome in Japan.

Although the overall prognosis of patients with Werner syndrome is improving, new opportunities for early diagnosis and treatment intervention are still needed. It has been suggested that the inclusion of the WRN gene in genetic analyses for early-onset diabetes, lipodystrophy, or dyslipidemia may offer an opportunity to diagnose patients with Werner syndrome long before the presentation of the full spectrum of symptoms and complications, enabling earlier interventions, including malignant neoplasm screenings and prevention of diseases listed in the clinical criteria [58]. Recently, novel senescent markers, ATP6V0D1 and RTN4, were shown to be increased in cells derived from patients with Werner syndrome, raising a possibility that they may serve as markers of disease progression [59]. Similarly, ribonuclease H2 subunit A (RNaseH2A) was shown to be downregulated in Werner syndrome cells as well as selected cancer cells [60]. Therefore, it may also be used as a diagnosis and severity marker. It was reported that patients with Werner syndrome have thinning of the retinal nerve fiber layer, ganglion cell complex, and choroidal thickness and the loss of visual field [61]. It may be useful for early diagnosis to have retinal and choroidal check-ups with the optical coherence tomography images when patients present with juvenile cataracts of unknown cause. For early detection of complications, genetic testing for complications, such as malignant neoplasms, may also be useful, when Werner syndrome is diagnosed. Early detection makes it possible to reduce risk factors such as smoking and to do early prevention and treatment.

Regarding renal protection, renin-angiotensin system inhibitors are hard to use, since many patients stopped their usage due to hyperkalemia. Additionally, sodium-glucose cotransporter 2 inhibitors are also difficult to use due to concern of sarcopenia in patients with Werner syndrome. However, a mineralocorticoid receptor antagonist, finerenone, may be a good candidate, since it is less likely to cause hyperkalemia.

Regarding intractable skin ulcers, treatment with a functional peptide SR-0379 was found to be safe, well-tolerated, and effective for leg ulcers in patients with Werner syndrome in clinical trials [62, 63].

Reportedly, p38 inhibitors reduced the accelerated cell senescence in primary fibroblasts from patients with Werner syndrome [64]. It is believed that in the near future, p38 inhibitors may be used in *in vivo* studies. Nicotinamide adenine dinucleotide (NAD) is important for the activation of sirtuin, a type of protein involved in regulating cellular processes including the aging and death of cells. NAD levels decrease in patients with Werner syndrome [65], therefore supplementation with NAD precursors may be effective. A clinical trial with nicotinamide riboside, a precursor of NAD, which may be effective for intractable ulcers, sarcopenia, and ASCVD, is investigating the safety and efficacy in patients with Werner syndrome [66]. Using state-of-the-art technologies, such as genome, transcriptome, and epigenome analyses [67], study of the generation of iPS cells from patients with Werner syndrome and the correction of the WRN gene by the CRISPR/Cas9-mediated methods [68] will create new treatments for patients with Werner syndrome.

In conclusion, this study clarified secular changes in multiple patients with Werner syndrome. The morbidity due to malignant neoplasms is high even in those of young age; therefore, it is necessary to carry out active and detailed screening examinations for malignant neoplasms. Renal function declines rapidly; therefore, evaluation based on BSA-uncorrected eGFRcre or eGFRcys and attention to the type and amount of drugs used are needed.

## MATERIALS AND METHODS

### Werner Syndrome Registry (patient registration system)

To reveal the disease profile and prognosis and to seek suitable medical intervention methods, the Werner Syndrome Registry was established. Based on the results of a nationwide primary survey, the patients were recruited to the registry from facilities with patients definitively diagnosed with Werner syndrome according to the diagnostic criteria [20].

The study complied with the ethical rules for human experimentation as specified in the Declaration of Helsinki. The study received approval from the Ethics Board of Chiba University on 27th July 2016 (approval number: 278) and from the Ethics Board of Kyoto University on 29th January 2020 (approval number: R2370). The study was registered at the UMIN Clinical Trial Registry (https://upload.umin.ac.jp/cgi-open-bin/ctr_e/ctr_view.cgi?recptno=R000034058) on 3rd November 2017 (ID: UMIN000029812). Written informed consent was obtained from patients before registration.

The key inclusion criteria for the registry were as follows: 1) patients with confirmed Werner syndrome based on the diagnostic criteria [20], and 2) patients who provided written informed consent prior to their participation in the registry. There were no exclusion criteria. The registry data included patient background (age at the time of onset, diagnosis, and registration; height, weight, BMI, abdominal circumference, and presence of consanguineous marriage), characteristic major signs and clinical symptoms, comorbidities (diabetes mellitus, impaired glucose tolerance, dyslipidemia, hypertension, fatty liver, cerebrovascular diseases, cardiovascular diseases, peripheral artery disease, foot amputation, and malignant neoplasms), the pattern of gene mutation, blood examination (blood cell count, biochemistry, liver and renal functions, and glucose and lipid profiles), visceral fat area, SMI, physical function (grip strength and walking speed), and treatment content. Renal function was evaluated using the following three indices: eGFRcre, BSA-uncorrected eGFRcre, and eGFRcys.

The calculation formula of each eGFR index was as follows:

(1) eGFRcre (mL/min/1.73 m^2^)

Male: eGFRcre (mL/min/1.73 m^2^) = 194 × Cr^−1.094^ × Age^−0.287^

Female: eGFRcre (mL/min/1.73 m^2^) = 194 × Cr^−1.094^ × Age^−0.287^ × 0.739

(2) BSA-uncorrected eGFRcre (mL/min) = eGFRcre × (BSA/1.73)

BSA can be calculated using the Du Bois method. BSA (m^2^) = Height (cm)^0.725^ × Weight (kg)^0.425^ × 0.007184.

(3) eGFRcys (mL/min/1.73 m^2^)

Male: eGFRcys (mL/min/1.73 m^2^) = {104 × CysC^−1.019^ × 0.996^Age^} −8

Female: eGFRcys (mL/min/1.73 m^2^) = {104 × CysC^−1.019^ × 0.996^Age^ × 0.929} −8

The datasheets were collected from the facilities annually. The collected data were subsequently inputted into the registration system Fountayn (previously named DATATRACK ONE) (NTT DATA, Tokyo, Japan) [9].

### Cross-sectional analysis and longitudinal analysis

The data were extracted on May 12, 2022. Data obtained at the registration time of each patient were analyzed through a cross-sectional analysis study model. For longitudinal analysis of the data in the Werner Syndrome Registry, the data at the time of initial registration and after one, two, and three years were compared in the patients with such data. For continuous variables, the paired t-test was used for items with a normal distribution, and the Wilcoxon signed-rank test was used for items with a non-normal distribution. For binary variables, Pearson’s chi-squared test was used for items with 20 patients and more, and Fisher’s exact test was used for items with less than 20 patients included. All data were analyzed with JMP pro 15 (SAS Institute, Cary, NC, USA). A *p*-value less than 0.05 was considered statistically significant.

The prevalence of malignant neoplasms and aspiration pneumonia was also investigated for the entire period from registration to three years later. Regarding malignant neoplasms, the presence or absence of malignant neoplasms, categorization of epithelial or non-epithelial neoplasms, and type of neoplasms were investigated.

Moreover, the association between renal function and leg ulcers/NSAIDs was also investigated. The relationship between renal function (eGFRcre, BSA-uncorrected eGFRcre, and eGFRcys) and both the presence or absence of leg ulcers and the use of NSAIDs were investigated using the baseline data.
